# Mutant phosphatidate phosphatase Pah1-W637A exhibits altered phosphorylation, membrane association, and enzyme function in yeast

**DOI:** 10.1016/j.jbc.2022.101578

**Published:** 2022-01-11

**Authors:** Yeonhee Park, Geordan J. Stukey, Ruta Jog, Joanna M. Kwiatek, Gil-Soo Han, George M. Carman

**Affiliations:** Department of Food Science and the Rutgers Center for Lipid Research, New Jersey Institute for Food, Nutrition, and Health, Rutgers University, New Brunswick, New Jersey, USA

**Keywords:** phosphatidate, diacylglycerol, triacylglycerol, Pah1, lipin 1, PA phosphatase, membrane, phospholipid, DAG, diacylglycerol, ER, endoplasmic reticulum, HAD, haloacid dehalogenase, PA, phosphatidate, PC, phosphatidylcholine, PE, phosphatidylethanolamine, PAP, phosphatidate phosphatase, PI, phosphatidylinositol, SC, synthetic complete, TAG, triacylglycerol

## Abstract

The *Saccharomyces cerevisiae PAH1*-encoded phosphatidate (PA) phosphatase, which catalyzes the dephosphorylation of PA to produce diacylglycerol, controls the bifurcation of PA into triacylglycerol synthesis and phospholipid synthesis. Pah1 is inactive in the cytosol as a phosphorylated form and becomes active on the membrane as a dephosphorylated form by the Nem1–Spo7 protein phosphatase. We show that the conserved Trp-637 residue of Pah1, located in the intrinsically disordered region, is required for normal synthesis of membrane phospholipids, sterols, triacylglycerol, and the formation of lipid droplets. Analysis of mutant Pah1-W637A showed that the tryptophan residue is involved in the phosphorylation-mediated/dephosphorylation-mediated membrane association of the enzyme and its catalytic activity. The endogenous phosphorylation of Pah1-W637A was increased at the sites of the N-terminal region but was decreased at the sites of the C-terminal region. The altered phosphorylation correlated with an increase in its membrane association. In addition, membrane-associated PA phosphatase activity *in vitro* was elevated in cells expressing Pah1-W637A as a result of the increased membrane association of the mutant enzyme. However, the inherent catalytic function of Pah1 was not affected by the W637A mutation. Prediction of Pah1 structure by AlphaFold shows that Trp-637 and the catalytic residues Asp-398 and Asp-400 in the haloacid dehalogenase-like domain almost lie in the same plane, suggesting that these residues are important to properly position the enzyme for substrate recognition at the membrane surface. These findings underscore the importance of Trp-637 in Pah1 regulation by phosphorylation, membrane association of the enzyme, and its function in lipid synthesis.

The *Saccharomyces cerevisiae PAH1*-encoded phosphatidate phosphatase (PAP) enzyme (1), which catalyzes the Mg^2+^-dependent dephosphorylation of phosphatidate (PA) to produce diacylglycerol (DAG) (2), plays a major role in governing whether cells utilize PA to synthesize the storage lipid triacylglycerol (TAG) or synthesize membrane phospholipids (1, 3, 4) (Fig. 1). In actively growing cells, PA is primarily converted to cytidine diphosphat-DAG for the synthesis of all major membrane phospholipids, whereas in cells entering stasis, PA is primarily converted to DAG for the synthesis of TAG (5, 6, 7, 8, 9). Mutants defective in the CDP-DAG–dependent pathway of phospholipid synthesis may utilize the DAG derived from the PAP reaction to produce phosphatidylcholine (PC) and/or phosphatidylethanolamine (PE) via the Kennedy pathway when cells are supplemented with choline and/or ethanolamine (5, 6). A defect in the dephosphorylation of PA, as caused by loss of Pah1 PAP activity (*e.g.*, *pah1*Δ and catalytic site mutations), results in a plethora of phenotypes (summarized in ref. (7)) that correlates with decreases in the synthesis of DAG and TAG as well as with increases in the PA level and phospholipid synthesis (1, 8, 10).

**Figure 1:**
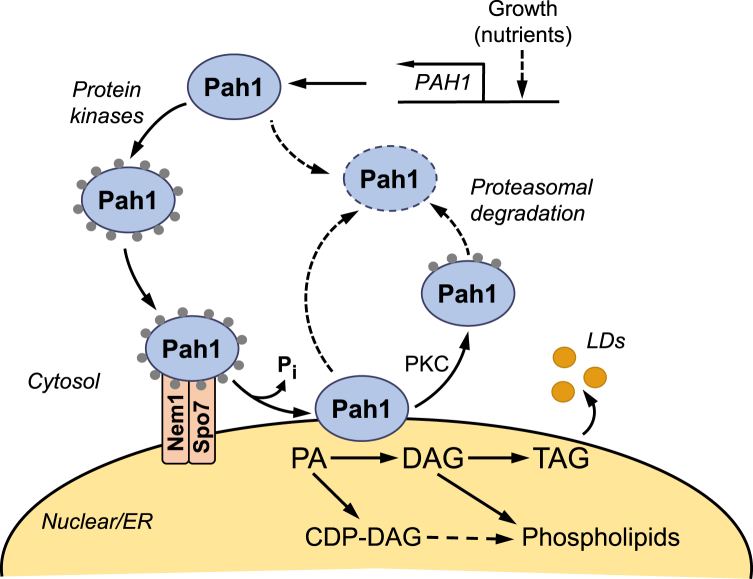
**Model for the regulation of Pah1 by phosphorylation, dephosphorylation, and proteasomal degradation.** Expression of *PAH1* is regulated during growth by nutrient status. After expression, Pah1 in the cytosol is phosphorylated by multiple protein kinases. Phosphorylated Pah1 (*small gray circles*) translocates to the nuclear/ER membrane through its recruitment and dephosphorylation by the Nem1–Spo7 protein phosphatase complex. Dephosphorylated Pah1 that is associated with the membrane catalyzes the conversion of PA to DAG, which is then acylated to form TAG that is stored in lipid droplets (*LDs*). Dephosphorylated Pah1 or protein kinase C (*PKC*)–phosphorylated Pah1 that is not phosphorylated at the target sites for Pho85-Pho80/Cdc28-cyclin B is degraded by the proteasome (indicated by the *dashed line arrows* and *ellipse*). DAG, diacylglycerol; PA, phosphatidate; TAG, triacylglycerol.

Pah1 PAP is a highly regulated enzyme that is controlled by genetic and biochemical mechanisms (7, 11, 12). It is transcriptionally regulated during growth by nutrient status; maximum expression coincides with the synthesis of TAG whereas reduced expression coincides with the synthesis of phospholipids (3, 13, 14). The PAP activity of Pah1 is stimulated by negatively charged phospholipids (15) but inhibited by positively charged sphingoid bases (16) and nucleotides (17). Phosphorylation is a major posttranslational modification for Pah1, and it is highly phosphorylated (18, 19, 20, 21, 22, 23, 24, 25, 26, 27, 28, 29, 30, 31) by multiple protein kinases (32, 33, 34, 35, 36, 37) (Fig. 2). Some of its phosphorylations are hierarchical in nature (*e.g.*, phosphorylation on one site affects phosphorylation on another site), whereas other phosphorylations occur on common sites by different protein kinases (7, 12). In general, phosphorylated Pah1, which is localized in the cytosol, is nonfunctional because it cannot directly access its substrate PA in the nuclear/endoplasmic reticulum (ER) membrane (19, 32, 33, 34). The Nem1–Spo7 complex (38) is a sole protein phosphatase that is responsible for the recruitment and dephosphorylation of Pah1 at the nuclear/ER membrane; this process is required for Pah1 to associate with the membrane for PAP activity (10, 19, 32, 33, 34, 38, 39, 40, 41, 42) (Fig. 1). In turn, the regulation of the Nem1–Spo7 complex impacts on the function of Pah1 (10, 43, 44, 45, 46, 47, 48). Phosphorylation has a protective effect on Pah1 against its degradation by the 20S proteasome, whereas its dephosphorylation makes it susceptible to the proteolytic degradation (49, 50). Overall, the reduced function of Pah1 due to its phosphorylation favors phospholipid synthesis at the expense of TAG synthesis, whereas the increased function of the enzyme by its dephosphorylation favors TAG synthesis at the expense of phospholipids synthesis (7, 12, 51). Pah1 is also subject to acetylation, a posttranslational modification that appears to be important for the enzyme function in TAG synthesis (52).

**Figure 2:**
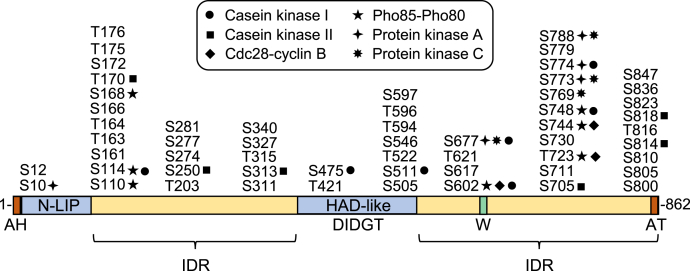
**Domains/regions and phosphorylation sites in Pah1.** The diagram shows the positions of the N-terminal amphipathic helix (*AH*), the conserved N-LIP and HAD-like catalytic domains, the conserved tryptophan (*W*) residue, the C-terminal acidic tail (*AT*), and the intrinsically disordered regions (*IDR*). The serine (*S*) and threonine (*T*) residues known to be phosphorylated are grouped at their approximate positions and marked for the responsible protein kinases. HAD, haloacid dehalogenase.

The domains/regions of Pah1 and its phosphorylation sites are shown in Figure 2. The conserved N-LIP and haloacid dehalogenase (HAD)-like domains (1, 53) are required for PAP activity (54, 55), and the N-terminal amphipathic helix is required for membrane interaction (39). In the membrane translocation of Pah1, its C-terminal acidic tail is required to interact with the Nem1–Spo7 protein phosphatase complex (40). The phosphorylation sites of Pah1, which are critical for regulating the location, activity, and stability of the enzyme (12), are concentrated in the intrinsically disordered regions (Fig. 2), which are located between the two conserved catalytic domains and at the C-terminal region (50).

A conserved tryptophan residue (Trp-637) of Pah1, which is located in the intrinsically disordered region, is required for the enzyme function in TAG synthesis, but not for its PAP activity *in vitro* (55). Because the function of Pah1 depends on its PAP activity, the functional loss of catalytically competent Pah1-W637A is an enigma that we addressed in this work. Here, we demonstrated that Trp-637 of Pah1 is important for the enzyme phosphorylation status and for its function in lipid synthesis and lipid droplet formation and that the functional defect of Pah1-W637A is not due to the impairment of its cytosol-to-membrane translocation or its inherent catalytic function. The structure of Pah1 predicted by AlphaFold (56) shows that Trp-637 and catalytic residues (*i.e.*, Asp-398 and Asp-400) almost lie in the same plane, suggesting that the proper alignment of the residues is important for the enzyme to recognize its substrate at the membrane surface *in vivo*.

## Results

### Cells expressing Pah1-W637A are defective in lipid synthesis and lipid droplet formation

**Figure 3:**
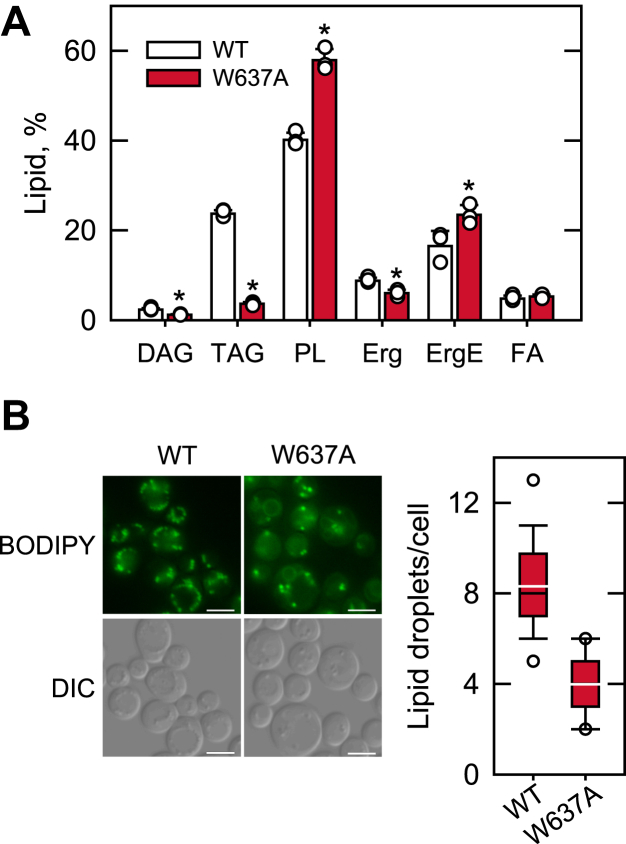
**Lipid composition and lipid droplet formation of cells expressing Pah1-W637A.** The *pah1*Δ cells were transformed with low-copy plasmids to express Pah1 or Pah1-W637A. *A*, the transformant cells were grown at 30 °C to the stationary phase in SC-Leu medium containing [2-^14^C]acetate (1 μCi/ml), and lipids were extracted from the radiolabeled cells. The extracted lipids were separated by TLC, subjected to phosphorimaging, and quantified by ImageQuant analysis. The levels of individual lipids were normalized to total chloroform-soluble fraction. The data are means ± SD. (error bars) from three separate experiments. The individual data points are also shown. ∗*p* < 0.05 *versus* WT. *B*, the transformant cells grown in SC-Leu medium to the stationary phase were stained with BODIPY 493/503 to visualize lipid droplets by fluorescence microscopy (*left*), and cellular lipid droplets were quantified (*right*) from ≥300 cells (≥4 fields of view). *White bar*, 2 μm. The *black* and *white lines* are the median and mean values, respectively, and the *white circles* are the outlier data points of the 5th and 95th percentile. DAG, diacylglycerol; DIC, differential interference contrast; TAG, triacylglycerol.

We previously showed that Trp-637 of Pah1 is required for its function in the synthesis of TAG (55). To further examine the importance of Trp-637 in Pah1 function, we analyzed the lipid composition of *pah1*Δ cells transformed with the W637A allele of *PAH1* on a low-copy plasmid. The transformant cells were grown with [2-^14^C]acetate to the stationary phase for the strongest effect of Pah1 PAP on lipid metabolism (1, 3, 57), and the radiolabeled lipids were extracted and analyzed by TLC. In *pah1*Δ cells expressing Pah1, the DAG and TAG levels accounted for 2.3 ± 0.3% and 23.7 ± 0.8%, respectively, of the total ^14^C-labeled lipids (Fig. 3*A*). However, the lipid levels of the cells expressing Pah1-W637A were 1.2 ± 0.1% and 3.6 ± 0.4%, which are 1.9- and 6.6-fold, respectively, lower than those of the WT control (Fig. 3*A*). In contrast to the levels of DAG and TAG, the phospholipid content of the cells expressing Pah1-W637A (57.9 ± 2.5%) was 1.4-fold higher than that of the cells expressing the WT enzyme (40.2 ± 1.5%) (Fig. 3*A*). These results are consistent with the previous finding that Pah1 controls the bifurcation of PA between TAG synthesis and phospholipid synthesis (1, 3, 57). Additionally, the amounts of ergosterol and ergosterol ester in cells expressing Pah1-W637A were 1.46-fold lower and 1.42-fold higher, respectively, when compared with the WT control (Fig. 3*A*). Fatty acid levels were not significantly affected by the W637A mutation. Among the distinct cellular phenotypes (7) displayed by the defect of Pah1 function is a significant reduction in the number of lipid droplets (8, 57). Accordingly, we examined the effect of the W637A mutation on Pah1 function in lipid droplet formation (Fig. 3*B*). Compared with *pah1*Δ cells expressing Pah1, which contained an average of eight lipid droplets per cell, those expressing Pah1-W637A contained an average of four lipid droplets per cell. The reduced number of lipid droplets caused by the W637A mutation was consistent with the mutational effect on the DAG and TAG levels.

### Translocation of Pah1-W637A to the membrane is dependent on the Nem1–Spo7 protein phosphatase complex

**Figure 4:**
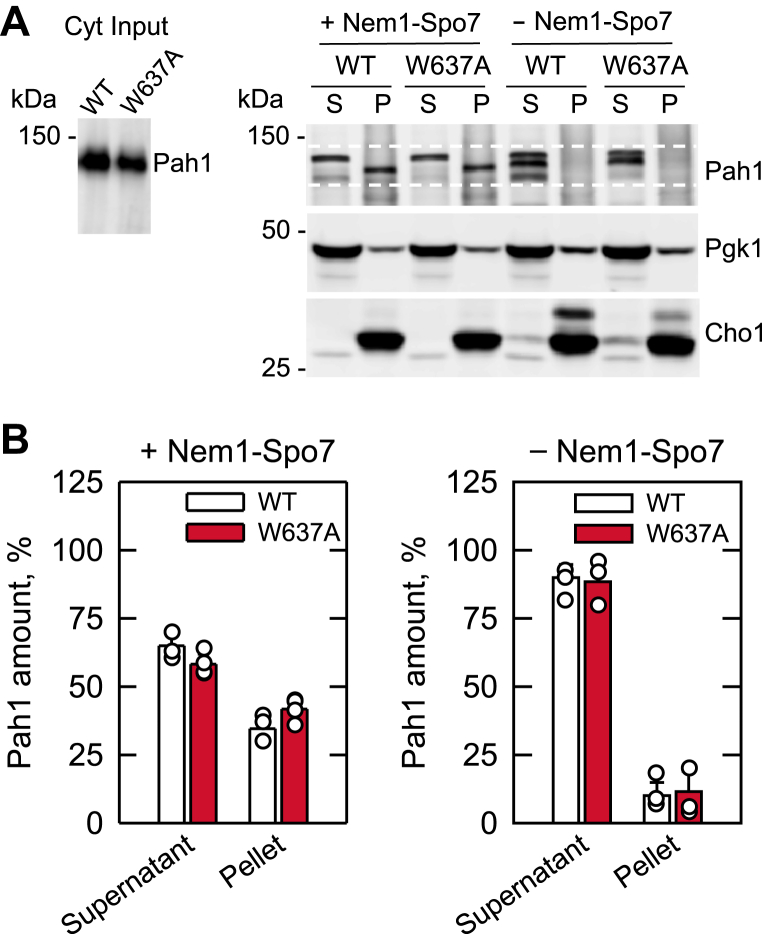
**Translocation of Pah1-W637A to the membrane.***A*, cytosol (*Cyt*) (15 μg) derived from *pah1*Δ *nem1*Δ cells expressing Pah1 or Pah1-W637A from a low-copy plasmid was incubated for 1 h at 30 °C with membranes (30 μg) derived from WT cells overexpressing the Nem1 and Spo7 (*+Nem1-Spo7 complex*) or *pah1*Δ *nem1*Δ cells (*−Nem1-Spo7 complex*). Following the incubation, the mixture was fractionated by centrifugation at 100,000*g* for 1 h at 4 °C. The pellet (*P*) fraction was resuspended in the same volume as the supernatant (*S*) fraction, and equal volumes of the fractions were subjected to SDS-PAGE using a 10% polyacrylamide gel. The proteins resolved in the polyacrylamide gel were transferred to a polyvinylidene difluoride membrane, which was cut for separately probing Pah1, Pgk1 (cytosol marker), and Cho1 (ER marker) with anti-Pah1, anti-Pgk1, and anti-Cho1 antibodies, respectively. The positions of Pah1, Pgk1, and Cho1 (the *upper* and *lower bands* are the protein kinase A-mediated phosphorylated and dephosphorylated forms of the protein, respectively (113)) and molecular mass standards are indicated. The *white dashed lines* are guides to show the range in the electrophoretic mobility of Pah1. The data shown are representative of four independent experiments. *B*, the relative amounts of supernatant- and pellet-associated Pah1 and Pah1-W637A were determined by ImageQuant analysis of four independent experiments ±SD (error bars). The immunoblot signals between the *white dashed lines* were used to quantify the amounts of Pah1. The individual data points are also shown.

We examined the translocation of Pah1-W637A from the cytosol to the membrane using an *in vitro* assay. In this assay, the cytosolic fraction containing the phosphorylated form of Pah1 or Pah1-W637A, which was prepared from cells lacking the Nem1–Spo7 complex (19), was incubated with the Pah1-free membrane fraction containing the overexpressed levels of the protein phosphatase. Following the incubation, the mixed subcellular fractions were separated again by centrifugation into the pellet (membrane) and soluble fractions. Immunoblot analysis showed that a significant amount (34 ± 4% and 41 ± 3.6%, respectively) of Pah1 and Pah1-W637A was detected from the membrane fraction containing the Nem1–Spo7 complex and that the membrane-associated proteins were faster in electrophoretic mobility (Fig. 4). However, in the absence of the protein phosphatase complex, only 10 ± 4% and 11 ± 7, respectively, of the WT and mutant proteins were present in the membrane fraction. These results demonstrated that Pah1-W637A properly translocates from the cytosol to the membrane in the Nem1–Spo7-dependent manner.

### Membrane localization of Pah1-W637A is dependent on the Nem1–Spo7 protein phosphatase complex

**Figure 5:**
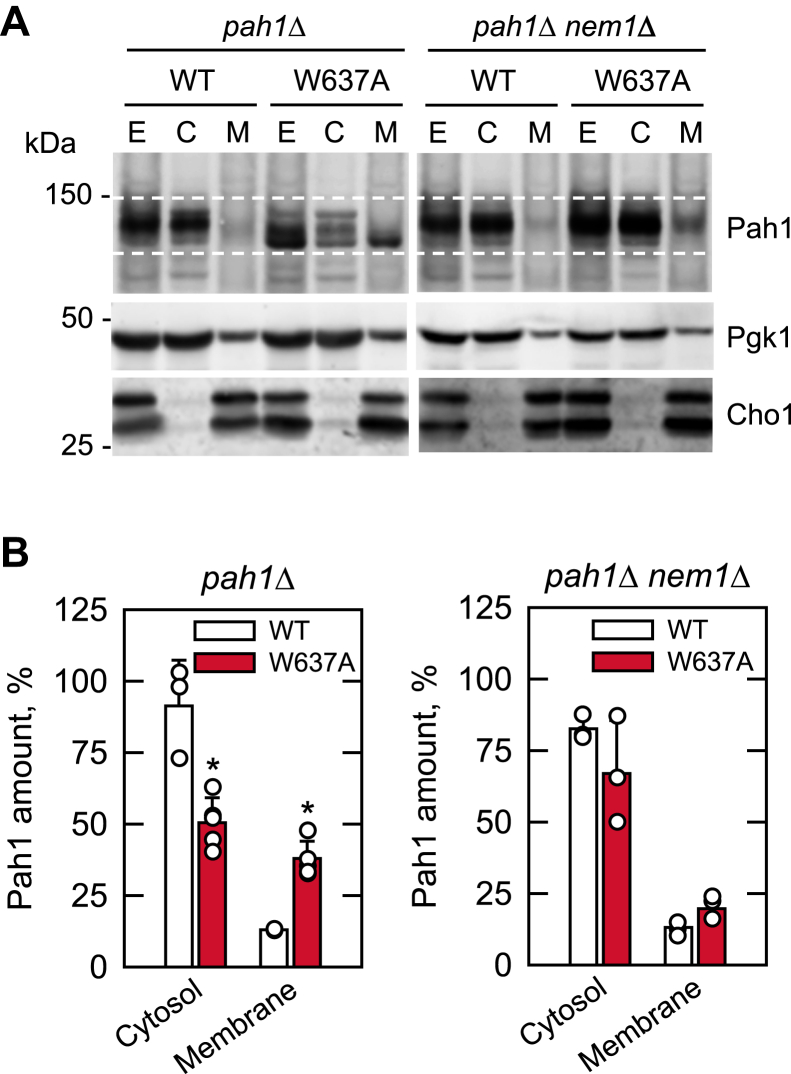
**Subcellular localization of Pah1-W637A.***A*, the *pah1*Δ and *pah1*Δ *nem1*Δ cells were transformed with low-copy plasmids to express Pah1 or Pah1-W637A. The transformants were grown at 30 °C to the mid-logarithmic phase in SC-Leu medium. Cell extracts (*E*) were fractionated into the cytosolic (*C*) and membrane (*M*) fractions by centrifugation at 100,000*g* for 1 h at 4 °C. The membrane fraction was resuspended in the same volume as the cytosolic fraction, and equal volumes of the fractions were subjected to SDS-PAGE using an 8% polyacrylamide gel. The proteins resolved in the polyacrylamide gel were transferred to apolyvinylidene difluoride membrane and probed with anti-Pah1 antibody. The cellular fractions were also subjected to SDS-PAGE using a 10% polyacrylamide gel. The proteins were transferred to polyvinylidene difluoride membrane, which was cut for separately probing Pgk1 (cytosol marker) and Cho1 (ER marker) with anti-Pgk1 and anti-Cho1 antibodies, respectively. The positions of Pah1, Pgk1, and Cho1 (the *upper* and *lower bands* are the protein kinase A-mediated phosphorylated and dephosphorylated forms of the protein, respectively (113)) and molecular mass standards are indicated. The *white dashed lines* are guides to show the range in the electrophoretic mobility of Pah1. The data shown are representative of at least three independent experiments. *B*, the relative amounts of cytosolic- and membrane-associated Pah1 and Pah1-W637A were determined by ImageQuant analysis of at least three independent experiments ±SD (error bars). The immunoblot signals between the *white dashed lines* were used to quantify the amounts of Pah1. The individual data points are also shown. ∗*p* < 0.05 *versus* WT. SC, synthetic complete.

Pah1 is mostly localized in the cytosolic fraction of the cell with a minor portion of the protein associated with the nuclear/ER membrane for its catalytic function (1, 39, 40, 42) (Fig. 1). We considered that the loss of Pah1 function by the W637A mutation in TAG synthesis and lipid droplet formation is rooted in a defective membrane association of the mutant protein *in vivo*. To address this possibility, cell extracts prepared from *pah1*Δ cells expressing Pah1, and Pah1-W637A were fractionated into the cytosolic and membrane fractions and examined for the protein levels by immunoblot analysis with anti-Pah1 antibody (Fig. 5). As expected (33), most of the Pah1 was shown in the cytosolic fraction with a small amount associated with the membrane fraction. In addition, the membrane-associated form of Pah1 was faster in electrophoretic mobility than its cytosolic form, indicating that it is the dephosphorylated form of the protein. Like the WT protein, Pah1-W637A associated with the membrane showed faster electrophoretic mobility than its cytosolic form. However, the mutant protein was more abundant (2.9-fold) in the membrane fraction than the WT protein and showed faster electrophoretic mobility. The increased association of Pah1-W637A with the membrane was also reflected by the reduction of its cytosolic level. These results indicated that W637A mutation of Pah1 does not impair but rather enhances its membrane localization.

The association of Pah1 with the membrane requires its dephosphorylation by the Nem1–Spo7 protein phosphatase complex in the nuclear/ER membrane (10, 38, 46). To determine whether the dephosphorylation-mediated membrane association of Pah1-W637A depends on the protein phosphatase complex, the mutant protein expressed in *pah1*Δ *nem1*Δ cells was examined for its protein level in the cytosolic and membrane fractions (Fig. 5). Immunoblot analysis of the subcellular fractions showed that Pah1 and Pah1-W637A are predominantly in the cytosol, and they are both slow in electrophoretic mobility. Interestingly, the WT and mutant proteins also exhibited an association with the membrane at a low level. However, their electrophoretic mobility was slower like that of the cytosolic forms. These results indicated that the dephosphorylated form of Pah1-W637A associates with the membrane in a manner dependent on the Nem1–Spo7 phosphatase complex.

### Pah1-W637A is catalytically competent

**Figure 6:**
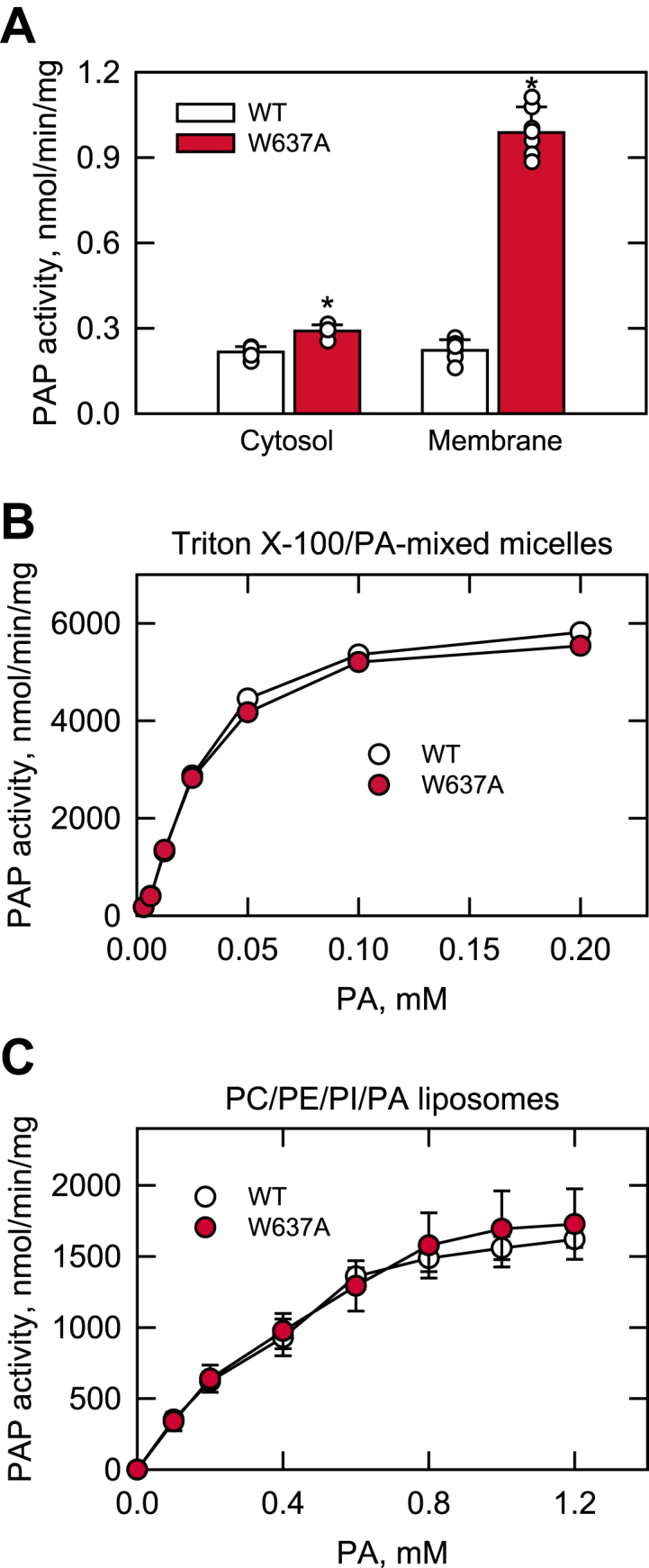
**PAP activity of Pah1-W637A.***A*, the *app1*Δ *dpp1*Δ *lpp1*Δ *pah1*Δ mutant cells were transformed with low-copy plasmids to express Pah1 and Pah1-W637A. Cell extracts were prepared from the transformants grown at 30 °C to the mid-logarithmic phase in SC-Leu medium and fractionated into the cytosolic and membrane fractions by centrifugation at 100,000*g* for 1 h at 4 °C. The membrane fraction was resuspended in the same volume as the cytosolic fraction, and equal volumes of the subcellular fractions were assayed for PAP activity by following the release of ^32^P_i_ from ^32^P-labeled PA in Triton X-100/PA-mixed micelles. The data shown are the average from three independent experiments ±SD (error bars). The individual data points are also shown. ∗*p* < 0.05 *versus* WT. *B* and *C*, unphosphorylated Pah1 and Pah1-W637A were expressed and purified from *E. coli*. The recombinant enzymes were assayed for PAP activity as a function of the indicated molar concentrations of PA using Triton X-100/PA-mixed micelles (*B*) and PC/PE/PI/PA liposomes (*C*). For the experiment shown in *B*, the molar ratio of Triton X-100 to PA was maintained at 10:1 (9.1 mol% PA). For the experiment shown in *C*, the molar concentration of PA was varied by increasing the amount of liposomes; the surface concentration of PA in the PC/PE/PI/PA liposome was 10 mol%. The data points represent the average of three experiments ±SD (error bars). The data shown in *B* and C were obtained with different enzyme preparations. PA, phosphatidate; PAP, phosphatidate phosphatase; SC, synthetic complete.

Because the cellular function of Pah1 requires its PAP activity (54), its functional loss by the W637A mutation was thought to be related to a defect in the membrane-associated enzyme activity. To address this possibility, we examined the PAP activity of Pah1 and Pah1-W637A expressed in *app1*Δ *dpp1*Δ *lpp1*Δ *pah1*Δ yeast cells (58), which lack all of the PAP enzymes encoded by the *APP1* (58), *DPP1* (59), and *LPP1* (60) genes. In an *in vitro* assay with Triton X-100/PA-mixed micelles, cell extracts containing Pah1-W637A were shown to catalyze the dephosphorylation of PA (55), indicating that it is catalytically competent. Pah1-W637A consists of two different forms in the cell, the phosphorylated form in the cytosol and the dephosphorylated one associated with the membrane. To better determine the catalytic competency of Pah1-W637A, its cell extract was separated into the cytosolic and membrane fractions. In PAP activity, the cytosolic fraction of Pah1-W637A was similar to that of the WT protein (Fig. 6*A*). In contrast, the membrane fraction of Pah1-W637A showed 4.4-fold higher PAP activity than that of the WT protein (Fig. 6*A*), which correlated with the elevated level of the mutant protein associated with the membrane (Fig. 5). The phosphorylated form of Pah1 in the cytosol is less active in PAP activity when compared with its dephosphorylated form associated with the membrane (19, 32, 33, 39). Consistent with the phosphorylation-mediated regulation, Pah1-W637A exhibited higher enzyme activity in its dephosphorylated state.

The dephosphorylated form of Pah1, which associates with the membrane, is relevant to its catalytic function for TAG synthesis. The completely dephosphorylated form of Pah1 can be represented by its unphosphorylated form. Accordingly, we examined unphosphorylated Pah1-W637A that was purified following its heterologous expression in *Escherichia coli*. The enzyme activity was measured with respect to the concentration of PA in Triton X-100/PA-mixed micelles (Fig. 6*B*) as well as in PC/PE/PI/PA liposomes (Fig. 6*C*). Triton X-100 forms a uniform mixed micelle with PA to provide an artificial surface for catalysis, whereas the PA-containing liposomes mimic a phospholipid bilayer (61, 62, 63). In the two assay systems, Pah1 and Pah1-W637A showed almost the same level of PAP activities that were dependent on the molar concentration of PA. These results showing the catalytic competency of unphosphorylated Pah1-W637A suggest that the W637A mutation of the enzyme does not affect its PAP activity in the dephosphorylated state on the membrane.

### The W637A mutation alters the endogenous phosphorylation of Pah1

**Figure 7:**
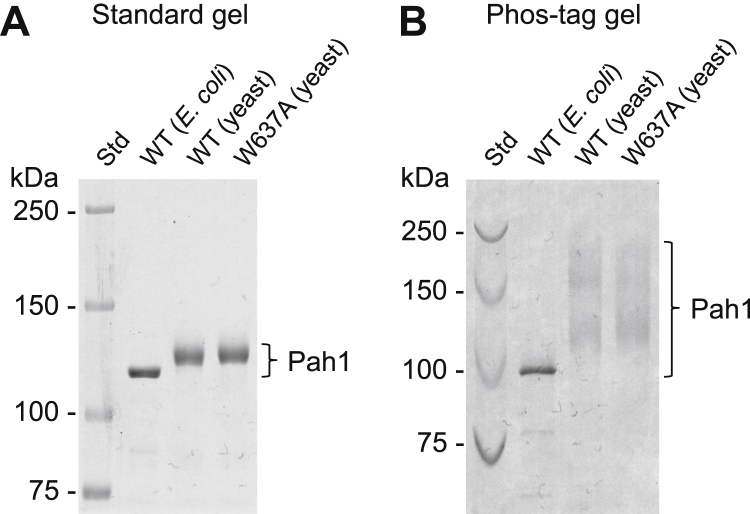
**Electrophoretic mobility of Pah1-W637A in SDS-PAGE.** The unphosphorylated and phosphorylated forms of Pah1 were purified from *E. coli* and yeast, respectively, as described in the “Experimental procedures.” Samples (0.5 μg) of the *E. coli*-expressed (unphosphorylated) Pah1 and yeast-expressed (phosphorylated) Pah1 and Pah1-W637A were subjected to SDS-PAGE using 6% polyacrylamide gels. *A*, in the absence of 20 μM Phos-tag and 100 μM MnCl_2_. *B*, in the presence of 20 μM Phos-tag and 100 μM MnCl_2_. The gels were stained with InstantBlue Coomassie protein stain. The positions of Pah1 and molecular mass standards (*Std*) are indicated.

The phosphorylation of Pah1 is required for its dephosphorylation-mediated membrane translocation, raising a question whether the *in vivo* phosphorylation of the protein is affected by the W637A mutation. To address this question, we expressed the protein A-tagged Pah1 and Pah1-W637A in *pah1*Δ *nem1*Δ cells, which maintain the proteins in the phosphorylated state due to a lack of the Nem1–Spo7 protein phosphatase complex (19) and purified them by IgG-Sepharose affinity chromatography. After the removal of protein A, the tag-free Pah1 and Pah1-W637A were further purified by anion exchange chromatography and size exclusion chromatography. We first examined the purified proteins for phosphorylation states by their mobility upon SDS-PAGE. Compared with the unphosphorylated form of Pah1, which was prepared following its heterologous expression in *E. coli*, the yeast-expressed Pah1 and Pah1-W637A showed a slower electrophoretic mobility (Fig. 7*A*), suggesting that they are phosphorylated proteins. To better assess the phosphorylation state, the proteins were resolved by Phos-tag SDS-PAGE (Fig. 7*B*). In the Phos-tag gel, the *E. coli*-expressed unphosphorylated Pah1 showed faster migration as a discrete band. In contrast, Pah1 and Pah1-W637A purified from yeast both showed a diffuse pattern of slower migration that differs in a great extent, indicating that they are highly and heterogeneously phosphorylated. Although Pah1-W637A was similar to Pah1 in electrophoretic mobility, it showed some minor difference in the heterogeneity of electrophoretic mobility.

**Figure 8:**
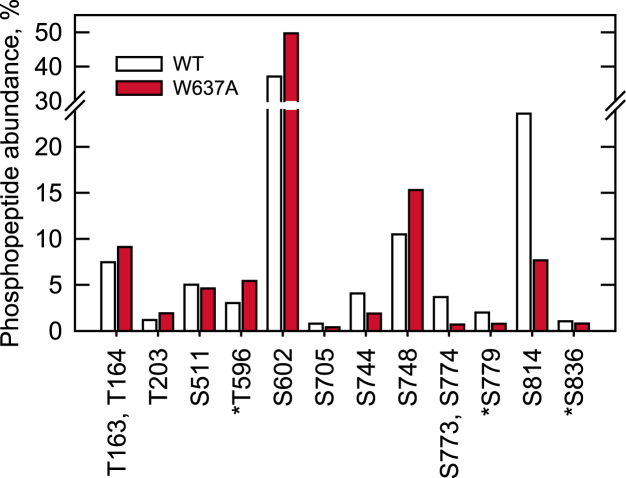
**Endogenous phosphorylation of Pah1-W637A.** Pah1 and Pah1-W637A were expressed and purified from yeast lacking the Nem1–Spo7 protein phosphatase complex. The proteins in SDS-polyacrylamide gel slices were extracted, reduced, alkylated, and digested with either trypsin, chymotrypsin, or Glu-C; the resulting peptides were analyzed as described in “Experimental Procedures.” Database search results from each digest were combined, and the abundance of phosphopeptides containing the indicated phosphorylation site(s) was estimated from intensities reported by Proteome Discoverer and expressed as a percentage of the intensities of all phosphopeptides identified for each protein (Table S1). The indicated amino acid residues are phosphorylation sites that were confidently assigned at ≥1% of the total phosphopeptide abundance. ∗, novel phosphorylation site.

To better understand the phosphorylation states of the yeast-purified Pah1 and Pah1-W637A, we determined their phosphorylation sites by LC-MS/MS (Fig. 8 and Table S1). This analysis confirmed many of the phosphorylation sites known in previous studies (18, 19, 20, 21, 22, 23, 24, 25, 26, 27, 28, 29, 30, 31) and identified some novel sites of phosphorylation (indicated by the *asterisks* in Fig. 8). Based on the abundance of phosphopeptides, Ser-602 (49%), Ser-814 (18%), and Ser-748 (8%) were the major sites of Pah1 phosphorylation *in vivo* (Fig. 8). Compared with Pah1, Pah1-W637A showed higher phosphorylation on Thr-203 (1.8-fold), Ser-277 (3-fold), Thr-596 (1.5-fold), Ser-602 (1.3-fold), and Ser-748 (1.2-fold) but lower phosphorylation on Ser-744 (2.7-fold), Ser-773 and/or Ser-774 (7-fold), Ser-779 (3-fold), and Ser-814 (3.6-fold) (Fig. 8 and Table S1), which are target sites for multiple protein kinases (Fig. 2).

### Limited proteolysis indicates that the W637A mutation does not have a major effect on Pah1 structure

**Figure 9:**
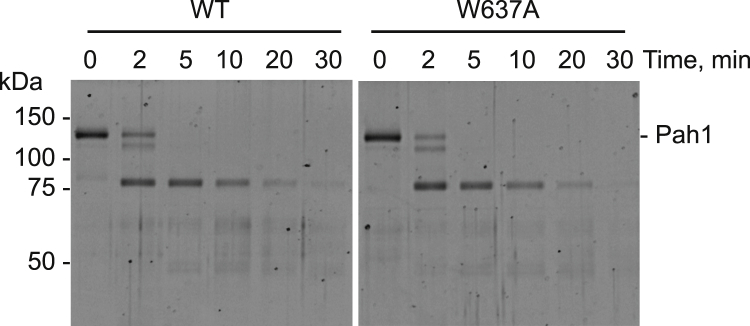
**Limited proteolysis of Pah1-W637A with chymotrypsin.** Samples (0.1 μg) of phosphorylated Pah1 and Pah1-W637A purified from yeast were incubated for the indicated time intervals at 23 °C with 50 ng of chymotrypsin in phosphate buffered saline (pH 7.4). The protein digests were separated by SDS-PAGE using 12% polyacrylamide gels and stained with SYPRO Ruby. The positions of full-length Pah1 and molecular mass standards are indicated.

Major changes of protein structure caused by amino acid substitutions are shown by different susceptibility to proteolytic degradation (64, 65). To examine whether the W637A mutation of Pah1 has an effect on its structure, we examined the degradation of Pah1-W637A with chymotrypsin. Pah1 and Pah1-W637A, purified following their overexpression in yeast cells lacking the Nem1–Spo7 protein phosphatase complex, were incubated with chymotrypsin for different periods of time and resolved by SDS-PAGE. The digestion patterns of Pah1 and Pah1-W637A in the limited proteolysis were very similar (Fig. 9), indicating that the W637A mutation does not have a major effect on the Pah1 structure.

## Discussion

Pah1 PAP is one of the key enzymes in yeast lipid metabolism as being required for the synthesis of TAG and the regulation of phospholipid synthesis (7, 12). As a result, yeast cells lacking the enzyme (1) or its catalytic activity (54) exhibit alterations in lipid metabolism and related cellular processes. For example, the *pah1*Δ mutant, which is defective in TAG synthesis, contains higher levels of phospholipids through increased availability of PA for phospholipid synthesis as well as through elevated gene expression of phospholipid biosynthetic enzymes (4, 10). In addition, it exhibits aberrant expansion of the nuclear/ER membrane (10), reduced lipid droplet formation (8), vacuole fragmentation (66), and weakened cell wall (67, 68). Moreover, it is defective in TORC1-mediated induction of autophagy (69) and growth on nonfermentable carbon sources (1, 54) and is more susceptible to fatty acid–induced lipotoxicity (57), heat (1, 10, 70, 71), cold (72), and oxidative stress (73). Ultimately, the mutant cells lacking the PAP activity have a shortened chronological life span (73) with apoptotic cell death in the stationary phase (57). As one might expect from the importance of the PAP enzyme, it is conserved in eukaryotic organisms (1, 53, 74, 75, 76, 77, 78, 79, 80, 81). In higher eukaryotes (*e.g.*, lipin proteins in mice and humans), the lack of the enzyme function is intimately associated with lipid-based syndromes (53, 82, 83, 84, 85, 86).

In prior work, we found that Trp-637 is required for Pah1 function to dephosphorylate PA to produce DAG for TAG synthesis in the cell (55). In this study, we further showed that cells expressing the W637A mutant protein (*i.e.*, Pah1-W637A) had additional alterations in lipid composition (*e.g.*, ergosterol and ergosterol esters) and were defective in lipid droplet formation. We addressed the possibility that the functional loss of Pah1-W637A is rooted in a defect in its translocation from the cytosol to the nuclear/ER membrane and/or a defect in its membrane-associated PAP activity. However, the W637A mutation does not impair the cytosol-to-membrane translocation or the inherent PAP activity of Pah1. Moreover, the membrane translocation of Pah1-W637A was dependent on the nuclear/ER-associated Nem1–Spo7 protein phosphatase complex, indicating its correct membrane translocation. The mutant enzyme exhibited an increased association with the membrane as a dephosphorylated form, and its overall PAP activity from the membrane was higher when compared with that of the WT enzyme. We considered that the increased membrane association of Pah1-W637A reflects its reduced proteasomal degradation (49, 50). In this case, the cytosolic level of the mutant enzyme is expected to be the same as that of the WT enzyme. However, the level of cytosolic Pah1-W637A was significantly lower than that of the WT enzyme, suggesting that the change is caused by the enhanced membrane translocation of the mutant enzyme.

The main subcellular location of Pah1 for its catalytic function is the nuclear/ER membrane (7). Yet, the analyses of the GFP-tagged Pah1 in diverse genetic backgrounds have shown punctate structures, revealing that a population of the enzyme associates with the nuclear vacuole junction, lipid droplet–ER contact sites, and the inner nuclear membrane (42, 87). Whether or not the W637A mutation of Pah1 affects its localization to these subcellular locations is unclear as this question was not addressed here.

Phosphorylated Pah1 is a substrate of the Nem1–Spo7 phosphatase complex. Accordingly, the phosphorylation state of Pah1-W637A seems related to its enhanced membrane association. In general, the mutant enzyme is more phosphorylated at the N-terminal region (Thr-203, Ser-277, Thr-596, and Ser-602) but less phosphorylated at the C-terminal region (Ser-744, Ser-773, Ser-774, Ser-779, and Ser-814). The decreased phosphorylation of sites at the C-terminal region correlated with increased membrane association, and this observation is reminiscent to that observed for the Pah1-7A mutant defective in phosphorylation by Pho85-Pho80 (32). However, it is unclear whether the mutational effects on phosphorylation at these regions are specifically responsible for enhanced interaction of the C-terminal acidic tail with Nem1–Spo7 (40) and/or of the N-terminal amphipathic helix with the membrane (39). The effects of the W637A mutation on the multiple phosphorylation of Pah1 are complex and difficult to explain in a definitive manner. Nonetheless, Pah1-W637A interacts with the membrane, and thus its defect in enzyme function *in vivo* cannot be attributed to its mislocalization.

Pah1-W637A is not defective in catalytic activity as determined by conventional *in vitro* PAP assays with the substrate in the Triton X-100/PA-mixed micelle or in the PC/PE/PI/PA liposome. Yet, the mutant protein is nonfunctional *in vivo* because it is defective in the production of DAG for TAG synthesis. Although the *in vitro* PAP assays indicate the catalytic competency of Pah1 (63), they did not provide insight into the role Trp-637 in Pah1 with the constraints of the native nuclear/ER membrane.

**Figure 10:**
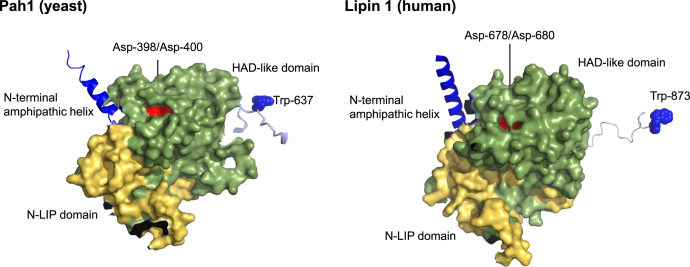
**Predicted structures of yeast Pah1 and human lipin 1.** The AlphaFold (56) structure predictions of yeast Pah1 and human lipin one were visualized with the PyMol program to highlight the conserved N-LIP and HAD-like domains and the N-terminal amphipathic helix. The positions of the Asp-398/Asp-400 and Asp-678/Asp-680 catalytic residues within the HAD-like domain, along with the conserved Trp-637 and Trp-873 residues of yeast Pah1 and human lipin 1, respectively, are indicated. HAD, haloacid dehalogenase.

Tryptophan residues are known to be involved in the protein–protein (88, 89) and protein–membrane (90, 91) interaction. Pah1 contains five tryptophan residues, four of which are found in the predicted structured regions (Trp-15 in the N-terminal amphipathic helix and Trp-388/Trp-390/Trp-420 in the HAD-like domain). In contrast, Trp-637 is predicted to locate in the intrinsically disordered region. The precise role of each tryptophan residue has not been determined, but we speculate that the first four are involved in membrane interaction and/or catalytic site positioning. The phosphorylated form of Pah1 as a substrate interacts with the Nem1–Spo7 phosphatase complex, and this substrate–enzyme interaction was not inhibited by the W637A mutation; Pah1-W637A was dephosphorylated by the phosphatase complex for its cytosol-to-membrane translocation. Whether or not Trp-637 mediates the interaction of Pah1 with an unknown protein required for its *in vivo* PAP activity is unclear. Most Pah1-interacting proteins identified by physical interaction studies are the plethora of protein kinases (32, 33, 34, 35, 36, 37, 92, 93, 94, 95) that are known or presumably responsible for the phosphorylation of the enzyme in the cytosol. Crystal structures for the conserved catalytic domains (N-LIP and HAD-like) of *Tetrahymena thermophila* Pah2 (96) and the membrane-binding M-Lip domain of mammalian lipin 1 (97) have been reported, but they provide no insight into the structural and/or functional role of Trp-637 in yeast Pah1 or of the corresponding tryptophan residue in its orthologous lipin proteins. The prediction of protein structure by AlphaFold (56) shows the positions of Trp-637 in yeast Pah1 and Trp-873 in human lipin one in relation to the positions of the N-LIP and HAD-like domains (Fig. 10). According to this model, Trp-637 and Trp-873 and their respective catalytic residues (*i.e.*, Asp-398 and Asp-400 in yeast Pah1 and Asp-678 and Asp-680 in human lipin 1) almost lie in the same plane, suggesting that the tryptophan residues are important to properly position the catalytic residues for substrate recognition at the membrane surface. This assertion, however, is only a prediction and may be an oversimplification of the binding and catalytic processes that occur *in vivo*. Additional studies are needed to address this hypothesis.

## Experimental procedures

### Reagents

Analtech was the source of silica gel GHL TLC plates. Lipids and polycarbonate filters used to prepare liposomes were purchased from Avanti Polar Lipids. Bio-Rad was the source of acrylamide solutions, protein molecular mass standards, protein assay reagents, and reagents for electrophoresis and immunoblotting. Anti-Pah1 (33) antibody was generated in rabbits at BioSynthesis, Inc. Clontech was the supplier of the carrier DNA used for yeast transformation. Difco Laboratories was the supplier of all growth media. InstantBlue Coomassie protein stain was purchased from Expedeon. GE Healthcare was the supplier of IgG-Sepharose, Q-Sepharose, polyvinylidene difluoride membrane, and the enhanced chemifluorescence substrate. Invitrogen was the source of DNA size ladders and mouse anti-Pgk1 antibodies (product number: 459250; lot number: E1161). Millipore-Sigma was the provider of leupeptin, pepstatin, ammonium molybdate, bovine serum albumin, chymotrypsin, Ponceau S stain, Triton X-100, and alkaline phosphatase–conjugated goat anti-mouse IgG antibodies (product number: A3562; lot number: SLBG1482 V). Scintillation counting supplies were purchased from National Diagnostics. New England Biolabs was the source of enzyme reagents for DNA manipulations, and PerkinElmer Life Sciences was the source of radiochemicals. Nickel–nitrilotriacetic acid agarose resin and kits for DNA gel extraction and plasmid purifications were obtained from Qiagen. Thermo Fisher Scientific was the supplier of Pierce strong anion exchange spin columns, alkaline phosphatase–conjugated goat anti-rabbit IgG antibody (product number: 31340, lot number: NJ178812), Pierce mass spectrometry grade proteases, Malachite green, BODIPY 493/503, and *S. cerevisiae* strain BY4741-*PAH1*-TAP. The Phos-tag AAL-107 reagent was obtained from Wako Pure Chemical Industries. All other chemicals used were reagent grade or better.

### Strains and growth conditions

The bacterial and yeast strains used in this study are listed in Table 1. *E. coli* strains DH5α and BL21(DE3)pLysS were used for the plasmid propagation and heterologous expression of His_6_-tagged Pah1, respectively. The *E*. *coli* cells were grown at 37 °C in lysogeny broth medium (1% tryptone, 0.5% yeast extract, 1% NaCl, pH 7.0); antibiotics (100 μg/ml ampicillin and 34 μg/ml chloramphenicol) were added to select for cells carrying plasmids. Solid growth media contained agar at the concentration of 1.5% and 2% for *E. coli* and yeast, respectively. The bacterial and yeast growth in liquid medium was measured by absorbance at 600 nm (A_600_) using a spectrophotometer. For overexpression of unphosphorylated Pah1 and Pah1-W637A, *E. coli* BL21(DE3)pLysS harboring plasmid pGH313 or plasmid pGH313-W637A was grown to A_600_ ∼ 0.5 in 500 ml of lysogeny broth medium containing ampicillin (100 μg/ml) and chloramphenicol (34 μg/ml); the protein expression was induced for 1 h at 22 °C with 1 mM isopropyl-*β*-D-thiogalactoside (1). The *S. cerevisiae pah1*Δ strains GHY57 (1) and SS1026 (10) were used for plasmid-directed expressions of Pah1 and Pah1-W637A. The *pah1*Δ *nem1*Δ strain SS1132, which lacks the Nem1–Spo7 protein phosphatase complex (33), was used to express the phosphorylated form of Pah1 for analysis of its subcellular translocation by dephosphorylation. BY4741-*PAH1*-TAP was used to PCR-amplify *PAH1*-TAP for the construction of pYES2-based plasmid pGH451 and its derivative pGH452. Standard methods were used to culture yeast cells at 30 °C (98, 99). Yeast transformants harboring a plasmid were grown in synthetic complete (SC) medium lacking a specific nutrient for plasmid selection. For overexpression of Pah1 and Pah1-W637A tagged with protein A, the SS1132 strain harboring plasmid pGH452 or plasmid pGH452-W637A was inoculated into 250 ml of SC-Ura/2% glucose medium to a final cell density of A_600 nm_ ∼ 0.1 and grown overnight to saturation. The saturated culture was harvested by centrifugation at 1500*g* for 10 min, and the cell pellet was resuspended (A_600_ ∼ 0.4) in 2 L of SC-Ura/1% raffinose/2% galactose medium and incubated for 14 h (A_600_ ∼ 1.0) with shaking at 250 rpm.

**Table 1:** Strains used in this study

Strain	Relevant characteristics	Source or reference
*E. coli*		
DH5α	F^−^ φ80d*lacZ*ΔΜ15Δ (*lacZYA*-*argF*)U169 *deoR rec*A1 *end*A1 *hsd*R17(*r*_k_^−^*m*_*k*_^+^) *pho*A *sup*E44 λ^−^*thi*-1 *gyr*A96 *rel*A1	(98)
BL21(DE3)pLysS	F^−^*ompT hsdS*_*B*_ (*r*_*B*_^−^*m*_*B*_^−^) *gal dcm* (DE3) pLysS	Novagen
*S*. *cerevisiae*		
W303-1A	*MAT***a***ade2-1 can1-100 his3-11,15 leu2-3112 trp1-1 ura3-1*	(116)
GHY57	*pah1*Δ*::URA3* derivative of W303-1A	(1)
GHY66	*pah1*Δ*::URA3 app1*Δ*::natMX4 dpp1*Δ*::TRP1/Kan*^*r*^*lpp1*Δ*::HIS3/Kan*^*r*^ derivative of W303–1A	(58)
RS453	*MAT***a** *ade2-1 his3-11,15 leu2-3112 trp1-1 ura3-52*	(117)
SS1026	*pah1*Δ*::TRP1* derivative of RS453	(10)
SS1132	*pah1*Δ*::TRP1 nem1*Δ*::HIS3* derivative of RS453	(33)
BY4741-*PAH1*-TAP	TAP-tagged Pah1 expressed in strain BY4741	Thermo Fisher Scientific

### DNA manipulations

The plasmids used in this study are listed in Table 2. Standard methods were used for isolation of chromosomal and plasmid DNA, for digestion and ligation of DNA and for PCR amplification of DNA (98, 99, 100). Plasmid transformations of *E*. *coli* (98) and yeast (101) were performed as described previously. Site-specific mutations were confirmed by DNA sequencing. Plasmid pGH315, which is a derivative of pRS415 (102), directs the low copy expression of Pah1 in yeast (33). The W637A derivative of plasmids pGH315 and pGH313 were constructed by PCR-mediated site-directed mutagenesis using appropriate primers as described previously (33). Plasmid pGH451 was generated by inserting the *PAH1*-TAP sequence, which was PCR-amplified from BY4741-*PAH1*-TAP DNA, into pYES2 at the KpnI and NotI sites. Plasmid pGH452 was derived from pGH451 by removal of a nucleotide sequence corresponding to the calmodulin-binding peptide of the TAP tag. Plasmid pGH452-W637A was derived from pGH452 by replacing the SacI/BamHI fragment of *PAH1* with that of pGH315-W637A. Plasmids YCplac111-*GAL1/10*-*NEM1*-PtA and pRS313-*GAL1/10*-*SPO7* were used for the overexpression of the Nem1–Spo7 protein phosphatase complex in strain RS453 (46).

**Table 2:** Plasmids used in this study

Plasmid	Relevant characteristics	Source or reference
pRS415	Single-copy number *E. coli*/yeast shuttle vector with *LEU2*	(102)
pGH315	*PAH1* inserted into pRS415	(33)
pGH315-W637A	*PAH1*(W637A) derivative of pGH315	(55)
pYES2	High-copy number *E. coli*/yeast shuttle vector with *URA3* and the *GAL1* promoter	Thermo Fisher Scientific
pGH451	*PAH1*-TAP in pYES2	This study
pGH452	Calmodulin binding peptide DNA sequence removed from the TAP tag in pGH451	This study
pGH452-W637A	*PAH1*(W637A) derivative of pGH452	This study
pET-15b	*E. coli* expression vector with N-terminal His_6_-tag fusion	Novagen
pGH313	*PAH1* coding sequence inserted into pET-15b	(1)
pGH313-W637A	*PAH1* (W637A) derivative of pGH313	This study
YCplac111-*GAL1/10*-*NEM1*-PtA	*NEM1*-PtA under control of *GAL1/10* promoter inserted in *CEN/LEU2* plasmid	(10)
pRS313-*GAL1/10*-*SPO7*	*SPO7* under control of *GAL1/10* promoter inserted in *CEN/HIS3* plasmid	(71)

### Radiolabeling and analysis of lipids

Steady-state labeling of yeast lipids with [2-^14^C]acetate was performed as described previously (103). Lipids were extracted from the yeast cells according to the method of Bligh and Dyer (104) as described by Fakas *et al.* (105). The extracted lipids were resolved by one-dimensional TLC on silica gel plates using the solvent system of hexane/diethyl ether/glacial acetic acid (40:10:1, v/v) (106). The resolved lipids were visualized by phosphorimaging using a Storm 860 Molecular Imager (GE Healthcare) and quantified by ImageQuant software using a standard curve of [2-^14^C]acetate. The identity of radiolabeled lipids was confirmed by comparison with the migration of authentic standards visualized by staining with iodine vapor.

### Analysis of lipid droplets

Lipid droplets in stationary phase cells were stained with the fluorescent dye BODIPY 493/503 (4, 45). The green fluorescence signal of the lipid droplets was observed under a Nikon Eclipse Ni-U microscope with the EGFP/FITC/Cy2/AlexaFluor 488 filter, recorded by the DS-Qi2 camera, and subjected to imaging analysis with the NIS-Elements BR software. The number of cellular lipid droplets was determined by examination of ≥300 cells.

### Preparation of subcellular fractions

All steps were performed at 4 °C. Yeast cultures were harvested in the exponential (A_600_ ∼ 0.5) and the stationary (A_600_ ∼ 3–4) phases of growth by centrifugation at 1500*g* for 5 min. The harvested cells were washed once with water and resuspended in 50 mM Tris-HCl (pH 7.5) buffer containing 0.3 M sucrose, 10 mM 2-mercaptoethanol, 1 mM Na_2_EDTA, 0.5 mM phenylmethylsulfonyl fluoride, 1 mM benzamidine, 5 μg/ml aprotinin, 5 μg/ml leupeptin, and 5 μg/ml pepstatin. The cell suspension was added with glass beads (0.5-mm diameter) and then subjected to five repeats of 1-min burst and 2-min cooling using a BioSpec Products Mini-Beadbeater-16 (107). The disrupted cells were centrifuged at 1500*g* for 10 min to separate cell extracts (supernatant) from unbroken cells and cell debris (pellet). The cell extract was centrifuged at 100,000*g* for 1 h to separate cytosolic (supernatant) from membrane fractions (pellet). The membranes were suspended in the cell disruption buffer without Na_2_EDTA. Protein concentration of the subcellular fractions was determined by the method of Bradford (108) using bovine serum albumin as the protein standard.

### Purification of Pah1

All steps were performed at 4 °C. The galactose-induced yeast cultures expressing protein A-tagged Pah1 and Pah1-W637A were harvested at 4 °C. The cell pellets (∼8 g) were resuspended in 16 ml of buffer I (50 mM Tris-HCl [pH 8.0], 150 mM NaCl, 1 mM EDTA, and Roche EDTA-free protease inhibitors) and lysed with a Mini-Beadbeater-16 (5 repeats of 1-min burst with 2-min cooling between bursts). The cell lysates were centrifuged at 1500*g* for 10 min at 4 °C, and the resulting supernatants were mixed with an equal volume of buffer I containing 2% Triton X-100 and centrifuged at 100,000*g* for 1 h at 4 °C. The supernatant was applied by gravity flow to a 0.5 ml IgG-Sepharose column equilibrated with buffer II (50 mM Tris-HCl [pH 8.0], 150 mM NaCl, 0.5 mM Na_2_EDTA, and 0.1% Triton X-100). The column was washed with 20-column volumes of the equilibration buffer and then incubated for 1 h at room temperature with 0.5 ml of buffer III (50 mM Tris-HCl [pH 8.0], 150 mM NaCl, 0.5 mM EDTA, 0.1% Triton X-100, 1 mM dithiothreitol) containing 75 units of tobacco etch virus protease. The protein A-free protein was eluted from the IgG-Sepharose resin with the chromatography buffer and then applied to a strong anion exchange spin column equilibrated with buffer IV (20 mM Tris-HCl [pH 8.0], 150 mM NaCl, and 10% glycerol). After the spin column was washed with buffer V (20 mM Tris-HCl [pH 8.0], 250 mM NaCl, and 10% glycerol), Pah1 was eluted from the column with buffer VI (20 mM Tris-HCl [pH 8.0], 500 mM NaCl, and 10% glycerol). The eluted enzyme, which is free of tobacco etch virus protease, was further purified by size exclusion chromatography using a Cytiva Superdex 200 Increase 10/300 GL column connected to a GE Healthcare AKTA Go Protein Purification System. The column was equilibrated and eluted with 20 mM Tris-HCl (pH 8.0) buffer containing 150 mM NaCl at a flow rate of 0.38 ml/min. The His_6_-tagged Pah1 and Pah1-W637A expressed in *E. coli* were purified by affinity chromatography with nickel–nitrilotriacetic acid—agarose (1) followed by ion exchange chromatography with Q-Sepharose (46) as described previously. The purified enzyme preparations were stored at −80 °C.

### Analysis of Pah1 phosphorylation by LC-MS/MS

Pah1 and Pah1-W637A expressed in yeast were analyzed for their endogenous states of phosphorylation by LC-MS/MS at the Center for Integrative Proteomics Research at Rutgers University, NJ. Polyacrylamide gel slices containing purified Pah1 were reduced with 10 mM dithiothreitol for 30 min at 60 °C, alkylated with 20 mM iodoacetamide for 45 min at room temperature, and digested overnight with 0.2 μg of trypsin at 37 °C (specificity, carboxyl side of Lys and Arg unless followed by Pro), chymotrypsin at 20 °C (specificity, carboxyl side of Tyr, Phe, Trp, and Leu), or Glu-C at 37 °C (specificity, carboxyl side of Asp and Glu). The resulting peptides were extracted twice with 5% formic acid, 60% acetonitrile and dried under vacuum. The peptides were subjected to LC-MS/MS using a Dionex UltiMate 3000 RSLCnano System (Thermo Fisher Scientific) interfaced with an Orbitrap Eclipse Tribrid Mass Spectrometer (Thermo Fisher Scientific). Each sample (1/20 of digests) was loaded onto an Acclaim PepMap 100 C18 HPLC silica trap column (Thermo Fisher Scientific). After washing for 5 min at 5 μl/min with 0.1% trifluoroacetic acid, the column was brought in-line with a nanoEase MZ Peptide BEH C18 column (130 Å 1.7 μm 75 μm × 250 mm) for LC-MS/MS analysis. Peptides were fractionated at 300 nl/min using a segmented linear gradient of 4 to 15% B in 5 min (where A: 0.2% formic acid, and B: 0.16% formic acid, 80% acetonitrile), 15 to 50% B in 50 min, and 50 to 90% B in 15 min. Solution B was then returned to 4% for 5 min before the next run. The scan sequence began with an MS1 spectrum (Orbitrap analysis, resolution 120,000, scan range from M/Z 275–1500, automatic gain control target 1E6, maximum injection time 100 ms). The top five (3 s) duty cycle scheme was used to determine the number of MS/MS scans performed for each cycle. Precursor ions of charges 2 to 7 were selected for MS/MS, and a dynamic exclusion of 60 s was used to avoid repeat sampling. Precursor ions were isolated in the quadrupole with an isolation window of 1.2 m/z, automatic gain control target 1E5, and fragmented with higher-energy collisional dissociation with a normalized collision energy of 30%. Fragments were scanned in Orbitrap with resolution of 15,000. MS/MS scan ranges were determined by the charge state of the parent ion; a lower limit was set to 110 m/z.

The raw data were analyzed with Proteome Discoverer Software (version 2.4.1.15) using the Sequest HT search engine against the UniProt yeast database (5983 proteins as of 05/18/2020), known Pah1 sequences, and a database composed of common laboratory contaminants (245 proteins). Precursor mass tolerance was set at 10 ppm, and fragment mass tolerance was set at 0.02 Da. Carboxyiodomethyl on cysteine was set as static modification, whereas acetylation, loss of methionine, and loss of acetylation plus methionine were set as dynamic modifications at the protein terminus. Up to two missed cleavages were allowed, while nonspecific cleavage was not permitted. Phosphorylation on serine and threonine as well as oxidation on methionine was set as dynamic modification. Percolator was used to validate results with strict target false discovery rate set to 0.01 and relaxed target rate set to 0.05 for both peptides and proteins. PhosphoRS was used to calculate phosphorylation sites possibilities (109). Precursor ion intensity was used to represent peptide and protein abundance. Peptides were grouped into protein groups using strict parsimony principle.

### SDS-PAGE and immunoblot analysis

SDS-PAGE (110) and immunoblotting (111, 112) with polyvinylidene difluoride membrane were performed by standard procedures. In Phos-tag SDS-PAGE, Phos-tag AAL-107 (20 μM) and MnCl_2_ (100 μM) were added to the resolving gels for analysis of the Pah1 phosphorylation state. The samples for immunoblotting were normalized to total protein loading; Ponceau S staining was used to monitor the protein transfer from the polyacrylamide gel to the polyvinylidene difluoride membrane. Rabbit anti-Pah1 (33) antibodies, rabbit anti-Cho1 antibodies (113), and mouse anti-Pgk1 antibodies were used at a concentration of 2 μg/ml. Alkaline phosphatase–conjugated goat anti-rabbit IgG antibodies and goat anti-mouse IgG antibodies were used at a dilution of 1:5000. Immune complexes were detected using an enhanced chemifluorescence substrate for alkaline phosphatase. Fluorescence signals from immunoblots were acquired by Storm 860 Molecular Imager (GE Healthcare), and the signal intensities were analyzed by ImageQuant TL software (GE Healthcare). A standard curve was used to ensure that the immunoblot signals were in the linear range of detection.

### PAP assays

In the radioactive assay, PAP activity was measured at 30 °C by following the release of water-soluble ^32^P_i_ from chloroform-soluble [^32^P]PA (10,000 cpm/nmol), which was enzymatically synthesized from DAG and [γ-^32^P]ATP with *E*. *coli* DAG kinase (107). In the colorimetric assay, the production of P_i_ from PA was measured with the malachite green–molybdate reagent (74, 114). The radioactive assay was used to measure PAP activity on the Triton X-100/PA-mixed micelle substrate (1), whereas the colorimetric assay was used to measure the enzyme activity on the phospholipid/PA liposome substrate (62). Triton X-100/PA (90:10 mol %) micelles were prepared by adding Triton X-100 to dried PA (61), whereas liposomes composed of PC/PE/PI/PA (45:30:15:10 mol %) were prepared by the extrusion method of MacDonald *et al*. (115) using a polycarbonate filter (100 nm) and a Mini-Extruder (Avanti Polar Lipids). The assay mixture contained 50 mM Tris-HCl (pH 7.5), 1 mM MgCl_2_, 10 mol% PA, and enzyme protein in a total volume of 100 μl (radioactive assay) or 10 μl (colorimetric assay). The enzyme assays were performed in triplicate, and all reactions were linear with time and protein concentration. A unit of PAP activity was defined as the amount of enzyme that catalyzes the production of 1 nmol of P_i_ per minute.

### Analyses of data

Statistical analyses were performed with SigmaPlot software. The *p* values <0.05 were taken as a significant difference.

## Data availability

Raw MS phosphorylation data and database search results are deposited in the MassIVE repository (https://massive.ucsd.edu/ProteoSAFe/static/massive.jsp) with the accession number MSV000087607. All other data are contained within the manuscript or the supporting information.

## Supporting information

This article contains supporting information .

## Conflict of interest

The authors declare that they have no conflicts of interest with the contents of this article.
