# Supplementary material for: Mutant phosphatidate phosphatase Pah1-W637A exhibits altered phosphorylation, membrane association, and enzyme function in yeast
Source: J Biol Chem. 2022 Jan 11;298(2):101578. doi: 10.1016/j.jbc.2022.101578 (PMC8819029; doi:10.1016/j.jbc.2022.101578)
Supplement: Supporting information [file mmc2.docx]

**­Mutant phosphatidate phosphatase Pah1-W637A exhibits altered phosphorylation, membrane association, and enzyme function in yeast**

Yeonhee Park, Geordan J. Stukey, Ruta Jog, Joanna M. Kwiatek, Gil-Soo Han and George M. Carman^*^

*From the Department of Food Science and the Rutgers Center for Lipid Research, New Jersey Institute for Food, Nutrition, and Health, Rutgers University, New Brunswick, New Jersey 08901*

Running title: *Trp-637 in Pah1 PA phosphatase*

^*^ For correspondence: George M. Carman, gcarman@rutgers.edu.

Supporting Information

Excel file with phosphorylation data
